# High frequency of IgE sensitization towards kiwi seed storage proteins among peanut allergic individuals also reporting allergy to kiwi

**DOI:** 10.1186/s12948-017-0073-4

**Published:** 2017-11-01

**Authors:** Jenny van Odijk, Sigrid Sjölander, Peter Brostedt, Magnus P. Borres, Hillevi Englund

**Affiliations:** 1 Dept of Respiratory Medicine and Allergology, Sahlgrenska Academy at Göteborg University, Göteborg, Sweden; 2 Internal Medicine and Clinical Nutrition, Sahlgrenska Academy at Göteborg University, Göteborg, Sweden; 3 R&D, ImmunoDiagnostic Division, Thermo Fisher Scientific, Uppsala, Sweden; 4 Department of Women’s and Children’s Health, Uppsala University, Uppsala, Sweden

**Keywords:** 2S albumin, 7S globulin, 11S globulin, IgE, Seeds, Storage proteins, Kiwi, Peanut

## Abstract

**Background:**

IgE sensitization to storage proteins from nuts and seed is often related to severe allergic symptoms. There is a risk of immunological IgE cross-reactivity between storage proteins from different species. The potential clinical implication of such cross-reactivity is that allergens other than the known sensitizer can cause allergic symptoms. Previous studies have suggested that kiwi seed storage proteins may constitute hidden food allergens causing cross-reactive IgE-binding with peanut and other tree nut homologs, thereby mediating a potential risk of causing allergy symptoms among peanut ant tree nut allergic individuals. The objective of this study was to investigate the degree of sensitization towards kiwi fruit seed storage proteins in a cohort of peanut allergic individuals.

**Methods:**

A cohort of 59 adolescents and adults with peanut allergy was studied, and self reported allergies to a number of additional foods were collected. Quantitative IgE measurements to seed storage proteins from kiwi and peanut were performed.

**Results:**

In the cohort, 23 out of the 59 individuals were reporting kiwi fruit allergy (39%). The frequency of IgE sensitization to kiwi fruit and to any kiwi seed storage protein was higher among peanut allergic individuals also reporting kiwi fruit allergy (*P* = 0.0001 and *P* = 0.01). A positive relationship was found between IgE levels to 11S globulin (r = 0.65) and 7S globulin (r = 0.48) allergens from kiwi and peanut, but IgE levels to 2S albumin homologs did not correlate. Patients reporting kiwi fruit allergy also reported allergy to hazelnut (*P* = 0.015), soy (*P* < 0.0001), pea (*P* = 0.0002) and almond (*P* = 0.016) to a higher extent than peanut allergic individuals without kiwi allergy.

**Conclusions:**

Thirty-nine percent of the peanut allergic patients in this cohort also reported kiwi fruit allergy, they displayed a higher degree of sensitization to kiwi storage proteins from both kiwi and peanut, and they also reported a higher extent of allergy to other nuts and legumes. On the molecular level, there was a correlation between IgE levels to 11S and 7S storage proteins from kiwi and peanut. Taken together, reported symptoms and serological findings to kiwi in this cohort of patients with concurrent allergy to peanut and kiwi fruit, could be explained by a combination of cross-reactivity between the 11S and 7S globulins and co-sensitization to the 2S albumin Act d 13.

## Background

Fruits, tree nuts and legumes like peanuts commonly cause allergic reactions in both children and adults affected by food allergy [1]. When prescribing an elimination diet, foods containing homologous proteins able to cause IgE cross-reactive reactions also need to be considered. As extensive food eliminations can lead to impaired quality of life, pin-pointing and exclusion of the clinically relevant foods only is of outmost importance [2]. There are common IgE epitopes on allergens from peanut and other plant foods [3], but the clinical relevance is not always known and both cross-reactivity and co-sensitization to these allergens must be considered [4, 5].

IgE sensitization to storage proteins has been associated with severe allergic reactions to several plant foods, especially tree nuts, peanuts and other legumes [6]. In 2014, storage proteins were identified also in seeds from green kiwi fruit, Act d 12 from the 11S globulin family and Act d 13 from the 2S albumin family [7]. Kiwi fruit is a common food allergen with allergic symptoms ranging from mild oral allergy symptoms to anaphylaxis [7, 8]. Evidence of IgE cross-reactivity between kiwi fruit storage proteins and homologs from nuts and legumes was presented in an in vitro study where binding of kiwi fruit specific IgE was inhibited by hazelnut, peanut and walnut protein extracts [9]. Recently, in silico studies also identified similar IgE-binding epitopes on Act c 12 from golden kiwi fruit seeds and other 11S globulins [10]. Considering the reports of concurrent peanut allergy and kiwi fruit allergy, in combination with the severity of allergic reactions caused by storage proteins, the objective of this study was to investigate the degree of IgE sensitization towards kiwi fruit seed storage protein in a cohort of peanut allergic individuals.

## Methods

In this study, a cohort of 59 peanut allergic adults living in the western area of Sweden (demographic data in Table 1) from a previously published study was studied. Detailed data about the group has been described previously [11]. In the original study, 74 patients were included, however limited by the amount of sera available the patient number was reduced to 59 in this study. Inclusion criteria were a known peanut sensitization (SPT positive and/or positive IgE to peanut and a convincing history of suspected peanut allergy). The participants completed a questionnaire about food allergies and specific questions about whether they suffered from allergies like birch or timothy grass pollen, and several foods like legumes, tree nuts and fruits [11]. Commercial ImmunoCAP reagents (Thermo Fisher Scientific, Uppsala, Sweden) were used for quantitative IgE-analyses to allergen extracts from peanut (f13) and kiwi (f84) and to recombinant Ara h 1, Ara h 2, Ara h 3, Ara h 8 and Act d 8. Analyses to Ara h 9 (LTP), Bet v 2 (profilin) and CCD were also performed. Experimental ImmunoCAP with native Act d 7S globulin, Act d 13 and Act d 12 purified from kiwi seeds were developed in house (Thermo Fisher Scientific, Uppsala, Sweden) as described previously [12]. The cut-off for positive IgE-level was defined as > 0.35 kU_A_/l. Group differences of IgE-levels were analyzed using Mann–Whitney test. Comparisons of frequency distributions between groups were analyzed by Fisher’s exact test (two tailed). Spearman’s rank correlation test was used to analyze the relationship between IgE concentrations. *P* values < 0.05 were considered significant.

**Table 1:** IgE sensitization to peanut and kiwi seed storage proteins, allergic symptoms after peanut ingestion and other allergies in a cohort of peanut allergic individuals (n = 59) whereof 39% also reported kiwi allergy

	All patients	Reporting kiwi allergy	Not reporting kiwi allergy	*P*
Demographic data				
N	59	23	36	
Median age (min–max)	23 (14–39)	23 (15–38)	23 (14–39)	
% female	69	74	67	
% sensitized (> 0.35 kU/l) in the group				
Any peanut storage protein	61	52	67	*0.04*
Any kiwi seed storage protein	41	52	33	*0.01*
Ara h 3 (11S globulin)	39	35	42	0.38
Act d 12 (11S globulin)	36	43	31	0.11
Ara h 1 (7S globulin)	49	39	56	*0.02*
Act d 7S (7S globulin)	36	43	31	0.11
Ara h 2 (2S albumin)	56	43	64	*0.005*
Act d 13 (2S albumin)	20	22	19	0.73
Ara h 8 (PR-10)	54	83	36	*<* *0.0001*
Act d 8 (PR-10)	59	83	44	*< 0.0001*
Peanut	90	87	92	0.36
Kiwi fruit	36	52	25	*0.0001*
% with reported allergy to				
Hazelnut	54	65	47	*0.015*
Soy	22	39	11	*<* *0.0001*
Lentil	14	17	11	0.31
Pea	32	48	22	*0.0002*
Almond	46	57	39	*0.016*

*P* values from Fisher’s exact test between frequencies in “with kiwi allergy” and “without kiwi allergy” groups, italics means *P* < 0.05

### Ethics approval

The study was approved by the Regional Ethical Review Board at Göteborg University and the collected personal data was treated according to the Swedish personal data act.

## Results

Of the 59 individuals with peanut allergy (age 14–39 years, median 23 years, Table 1), 23 (39%) reported to suffer from allergy symptoms when eating kiwi. Of these 23, 12 (52%) displayed IgE sensitization to kiwi fruit and 12 (52%) to one or more of the kiwi seed storage proteins (Table 1). The frequency of IgE sensitization to kiwi fruit and to any kiwi storage protein were higher among patients reporting kiwi allergy than those who did not (*P* = 0.0001 and *P* = 0.01, Table 1).

The opposite was found for peanut storage proteins where the frequency of sensitization to one or more peanut storage protein was higher among the individuals not reporting kiwi fruit allergy (67% vs 52%, *P* = 0.04). For individual components, a higher degree of sensitization to Ara h 1 and Ara h 2 was noted among the non-kiwi fruit allergic (*P* = 0.02 and *P* = 0.005, Table 1).

**Fig. 1:**
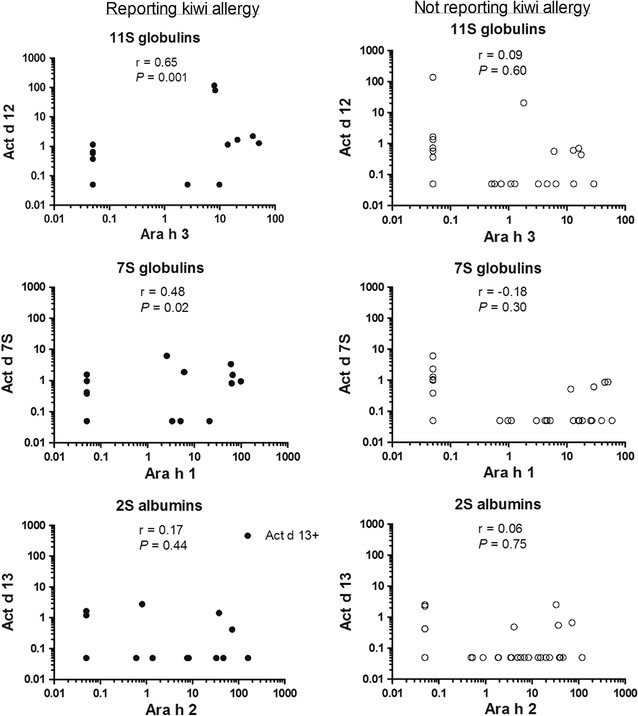
Correlations between IgE concentrations to 11S globulin, 7S globulin and 2S albumin from peanut (on X-axis) and kiwi seeds (on Y-axis) in sera from peanut allergic individuals reporting (closed circle) and not reporting (opened circle) kiwi allergy. Using Spearman’s rank test, significant correlations were found between 11S globulins and 7S globulins from kiwi and peanut. *P* and r values for each correlation are given in the graphs

IgE levels to kiwi fruit were higher among the patients reporting kiwi allergy than those who did not (mean level 3.0 vs 0.3 kU/l, *P* = 0.009), however IgE levels towards whole peanut, peanut storage proteins and kiwi seed storage proteins did not differ between groups (data not shown). IgE concentrations to homologous 11S globulins (Act d 12 and Ara h 3) and 7S globulins (Act d 7S globulin and Ara h 1) from peanut and kiwi displayed a positive correlation, but the 2S albumins (Act d 13 and Ara h 2) did not (Fig. 1). For individual patients with concurrent peanut and kiwi allergy, there was no clear pattern regarding primary sensitizing allergens. In seven patients a general pattern of higher IgE-levels to peanut storage proteins was observed, while in five patients the levels of IgE to kiwi storage proteins were higher (Table 2).

**Table 2:** Individual data of patients included in the study

Patient#	IgE analyses, kU/l													Age	Gender	Reported foods to cause allergy symptoms					
	% of patients > 0.35 kU/l for each allergen															% reporting allergy symptoms				Almond	Kiwi
	Peanut	Kiwi	Ara h 1	Ara h 2	Ara h 3	Act d 12	Act d 7S	Act d 13	Ara h 8	Act d 8	Ara h 9	Bet v 2	CCD			Hazelnut	Soy	Lentils	Pea		
1	1.8	2.5	0	0	0	0.4	0.4	0	59.3	12.7	0	0	0	19	F	x				x	x
2	173	9.5	99.0	73.5	51.2	1.3	0.9	0.4	12.7	3.9	0	0	0	25	F	x	x	x	x		x
3	27.7	13.3	2.6	0	7.9	115	6.2	0	47.5	35.9	0	0	0	15	M	x	x	x	x	x	x
4	2.3	0.8	0	1.4	0	0	0	0	7.7	0	0	0	0	23	F	x	x		x	x	x
5	17.5	18.8	6.0	0	8.3	78.9	1.9	1.7	20.4	2.1	0.6	0	0	15	F			x			x
6	0	0	0	0	0	0	0	0	0.6	0.4	0	0	0	15	M	x				x	x
7	0.5	0	0	0	0	0	0	0	2.7	2.4	0	0	0	26	F	x		x	x		x
8	169	5.4	61.0	33.3	39.6	2.2	3.4	0	0	1.1	0	0	0	19	M		x		x	x	x
9	0.9	0	0	0.6	0	0	0	0	2.8	0.4	0	0	0	28	F	x				x	x
10	0.6	0	0	0	0	0	0	0	2.4	2.3	0	0	0	35	F	x				x	x
11	9.9	3.6	0	0	0	0.6	1.0	0	64.7	63.0	0.4	0	0	17	F	x				x	x
12	64.4	1.4	21.0	47.2	9.8	0	0	0	6.7	3.1	0	0	0	37	F	x	x		x		x
13	16.8	0	3.4	7.7	0	0	0	0	2.9	5.1	0	0.8	0	19	F						x
14	169	0.4	66.3	160	20.8	1.7	1.5	0	5.8	1.5	0	0	0	15	F		x		x		x
15	22.5	0	5.1	8.4	2.6	0	0	0	0	0	0	0	0	25	F		x		x		x
16	112	0	63.1	37.7	13.9	1.1	0.8	1.4	1.4	1.9	0	0	0	24	F						x
17	0	0	0	0	0	0	0	0	0	0	0	0	0	20	M				x		x
18	4.7	0	0	0	0	0	0	0	35.7	58.2	0	0	0	18	M					x	x
19	0.5	2.1	0	0	0	0	0	0	5.9	5.5	0	0	0	33	F	x			x	x	x
20	0	0.5	0	0	0	0	0	1.2	3.4	3.3	0	0	0	24	F	x	x		x		x
21	1.9	10.0	0	0	0	0	0.4	0	42.6	33.7	0	0	0	26	F	x				x	x
22	0.8	0	0	0.8	0	0.6	1.5	2.8	0	0	0	0	0	38	M	x				x	x
23	0.7	0	0	0	0	1.1	0	0	5.4	0.8	0	0	0	23	F	x	x			x	x
	87	52	39	43	35	43	43	22	83	83	9	4	0		74	65	39	17	48	57	100
24	8.1	0	5.2	5.0	0	0	0	0	0	0	0	0	0	30	M					x	
25	7.2	0	0	1.9	0	0	0	0	0	0	0	0	0	15	M						
26	195	0	45.0	72.2	16.0	0.7	0.8	0.7	0	0	0	2.0	0	17	M	x				x	
27	3.0	0	3.0	0.5	0	0	0	0	1.0	0.9	0	0	0	18	F						
28	77.3	0	26.9	40.6	6.5	0	0	0	0	0	0	0	0	17	F						
29	5.3	0	1.0	1.9	0	0	0	0	0	0	0	0	0	19	F	x					
30	1.0	0	0	0.5	0	0	0	0	4.0	4.6	0	0	0	24	F	x					
31	70.9	0	39.5	15.3	13.1	0	0	0	0	0	0	0	0	18	F	x					
32	195	0	59.8	38.9	29.1	0	0	0	0	0	0	0	0	18	F						
33	2.2	1.4	0	0	0	1.6	2.3	0	0	0	0	0	1.3	27	F						
34	0.5	0	0	0	0	0	0	0	0	12.4	0	0	0	14	M	x				x	
35	1.1	0	0	0.9	0	0	0	0	0	0	0	0	0	16	M						
36	1.5	0.6	0	0	0	0.6	1.3	0	0	0	0	0	0.8	14	F						
37	9.4	0	4.5	4.1	0	0	0	0.5	0	0	0	0	0	15	M	x					
38	0	0	0	0	0	0.7	1.0	2.5	0	0	0	0	0	18	F	x				x	
39	4.3	0.6	0	0	1.8	20.4	1.0	2.3	2.1	3.5	0	0.9	0	22	F						
40	0.6	0.5	0	0	0	0	0	0	2.0	3.8	0.9	0	0	30	F	x					
41	19.5	0	15.7	5.7	0.6	0	0	0	0	0	0	0	0	25	M	x	x		x	x	
42	13.1	0	1.1	8.4	0.5	0	0	0	0	0	0	0	0	37	F						
43	115	1.0	50.8	36.7	12.9	0.6	0.9	0.5	5.1	4.1	11.3	1.0	1.0	16	M	x	x	x	x	x	
44	35.3	0	18.5	13.6	1.3	0	0	0	0	0	0	0	0	38	F	x	x	x	x	x	
45	89.7	0	25.7	19.9	0.7	0	0	0	0	0	0	0	0	19	M					x	
46	14.5	0	4.2	3.6	1.1	0	0	0	1.4	0.9	0	0	0	34	F				x		
47	72.2	0	16.3	46.5	3.2	0	0	0	0	0	0	0	0	34	M		x	x	x		
48	16.4	0	4.5	6.8	0	0	0	0	12.8	22.6	0	0	0	28	F						
49	0	1.6	0	0	0	0	0	0	4.4	8.7	0	0	0	29	F			x	x		
50	149	0.7	29.1	119	17.6	0.4	0.6	0	7.3	7.1	0	0	0	28	F				x		
51	0.8	0.6	0	0	0	0	0	0	3.0	2.0	0	0	0	27	M	x			x	x	
52	41.7	0	12.9	23.8	4.6	0	0	0	0	0	0	0	0	39	F	x					
53	19.5	1.3	0	0	0	135	6.1	0.4	14.2	1.0	1.2	0	0.8	22	F						
54	61.8	0	11.7	33.0	6.0	0.6	0.5	2.5	2.8	0.7	0	0	0	23	F	x				x	
55	0.9	0	0	0	0	1.3	1.0	0	0	0.5	0	0	0	22	M	x				x	
56	0.4	0	0	0	0	0	0	0	0	0	0	0	0	28	F					x	
57	0.5	0	0	0	0	0	0	0	1.6	0.5	1.2	0	0	29	F	x				x	
58	9.4	0	0.7	3.5	0	0	0	0	0	0	0	0	0	23	F						
59	0	0	0	0	0	0.4	0.4	0	0	1.6	0	0	0	25	F	x				x	
	92	25	56	64	42	31	31	19	36	44	11	8	11	14	67	47	11	11	22	39	0

For the PR-10 proteins, Ara h 8 and Act d 8, a higher degree of sensitization towards these proteins (*P* < 0.0001 for both Act d 8 and Ara h 8, Table 1), was demonstrated in the group reporting kiwi allergy. Additionally, the IgE levels towards these proteins were higher in the group reporting kiwi allergy (*P* = 0.004 for Act d 8 and *P* < 0.0001 for Ara h 8). For all patients, a low degree of sensitization to Ara h 9 (LTP), Bet v 2 (profilin) and CCD was observed and levels did not differ significantly between groups (Table 2).

The group reporting kiwi fruit allergy also reported significantly higher frequencies of allergies to other foods including hazelnut (*P* = 0.015), soy (*P* < 0.0001), pea (*P* = 0.0002) and almond (*P* = 0.016), but not for lentils (*P* = 0.31) (Table 1).

## Discussion

IgE sensitization to storage proteins have been associated with severe clinical reactions for several nuts, seeds, legumes and now recently also for fruit seeds [5–7, 9]. In this study of peanut allergic adolescents and adults, more than a third reported allergy symptoms when eating kiwi and more than half of them presented with IgE to one or more kiwi seed storage proteins, underlining the importance of also including seeds from fruits in the diagnostic work-up of peanut allergic individuals.

The frequency of concurrent kiwi and peanut allergy in this study, is in line with studies by Lucas et al. and Sirvent et al. [9, 13] studying peanut allergy among kiwi allergic subjects. In the study by Lucas et al., it was noted that more than half of the children with kiwi fruit allergy experiencing severe symptoms also reported peanut and tree nut allergies [13]. A number of these children reacted to their first known exposure of kiwi fruit indicating that they were primarily sensitized to a cross reacting allergen from another source like peanut and/or tree nuts [13]. This IgE cross-reactions was confirmed in vitro in a study by Sirvent et al., where peanut, almond, hazelnut and walnut inhibited the binding of IgE to both Act d 12 and Act d 13 [9] in sera from Spanish adults diagnosed with kiwi allergy, implying that the proteins share common epitopes. Existence of shared epitopes on homologous 11S globulin proteins was also noted in another in silico study by Barre et al. [10].

On the molecular level, there was some correlation between IgE-levels specific for the 11S and 7S globulins from kiwi and peanut, but not for the 2S albumins. The results are supported by a study by Ballabio et al., where 11S globulins were demonstrated to be the protein which mediated the highest degree of IgE cross-reactivity between proteins from several legumes and peanut [4]. Similar to the results in this study, IgE antibodies towards 2S albumins from hazelnut (Cor a 14) was demonstrated to not cause cross-reactive allergic reactions to the homologous protein from peanut (Ara h 2), although peanut allergy was common among hazelnut allergic individuals [5].

The peanut allergic patient’s reporting concurrent kiwi fruit allergy, to a higher extent also reported allergies to other storage protein containing foods than peanut allergic patients without kiwi allergy. This may indicate the presence of cross-reactive IgE antibodies to these other foods, but could also be explained by these individuals being more susceptible to develop new allergies in general. The presence of a general cross-reactive pattern is strengthened by the observation that the patients with concurrent allergy to kiwi fruit and peanut, additionally to displaying a higher frequency of cross-sensitization to 11S and 7S globulins, also presented with a high degree of cross-reactivity to PR-10 proteins. The group of peanut allergic patients *not* reporting kiwi allergy in this study displayed a higher frequency of peanut storage protein sensitization, higher degree of sensitization to the peanut allergy marker Ara h 2, and less symptoms to other nuts and legumes.

Evidence of immunological cross-reactivity between homologous storage proteins in nuts, seeds and legumes is well-known. In addition, there are several reports also for allergy symptoms among peanut and tree nut allergic individuals caused by cross-reactions to storage proteins from fruit and plant seeds [14–20]. In this study, this nut and fruit seed cross-reactivity is further highlighted by the findings from patients reporting allergy to both peanut and kiwi. Storage proteins have stable IgE-binding epitopes that often are resistant to both heating and gastrointestinal processing [5, 14]. Seeds from fruits can be ingested both intentionally and accidentally, as seed storage proteins can leak during food processing leading to unintentional contamination of for example fruit juices [15]. From a safety perspective for the patient with a peanut or tree nut allergy, it is important to investigate also the risk of allergic reactions caused by cross-reactive IgE antibodies to fruit storage proteins, especially since fruit seeds can present as hidden allergens.

The major limitation of this study is the use of data from self-reported, and not food challenge proven, kiwi fruit allergy. The major kiwi fruit allergen—Act d 1—is abundant in kiwi pulp and in kiwi fruit protein extract, and sensitization to Act d 1 is a marker of severe kiwi allergy [8]. In addition, sensitization towards kiwi seed storage proteins has been reported as a marker of kiwi allergy [7]. Of the 23 individuals reporting allergy symptoms in this study, a vast majority (n = 15, 65%) were sensitized either to kiwi fruit extract or kiwi seed storage proteins, supporting the validity of their self-reported kiwi allergy symptoms.

## Conclusions

The frequency of reported allergy to kiwi fruit was high in a cohort of Swedish adolescents and adults with peanut allergy. Further, in the group of patients that reported concurrent symptoms to kiwi fruit, a majority displayed IgE-reactivity to storage proteins from kiwi seeds and a correlation between IgE-levels to 11S and 7S globulins and PR-10 proteins from kiwi and peanut. These patients also reported a high frequency of symptoms to other nuts and legumes. In the group of peanut allergic patients not reporting kiwi allergy, the individuals displayed a higher frequency of peanut storage protein sensitization, especially to Ara h 2, and presented with fewer symptoms to other nuts and legumes. The results implicate the presence of at least two phenotypes of peanut allergic individuals in this cohort. One group with a broader cross-sensitization profile and having co-existing symptoms to both kiwi and other plant foods as well as co-sensitization to kiwi Act d 13, and a second group with a strong peanut sensitization to the major allergen Ara h 2 and less symptoms to other plant foods. The clinical relevance and implication of these results remains to be elucidated.

## Abbreviations

IgE: immunoglobulin E
kU/l: kilounits of allergen-specific IgE per liter
